# Cellular Senescence in Blood Cells: A Link Between Metabolic Syndrome, Inflammaging and Cardiometabolic Disease

**DOI:** 10.3390/cells15171615

**Published:** 2026-09-05

**Authors:** Katerina Gioti, Maria Trapali, Irene Belouka, Dimitrios Chaniotis, Apostolos Beloukas

**Affiliations:** Department of Biomedical Sciences, School of Health Sciences, University of West Attica, 12243 Athens, Greece

**Keywords:** metabolic syndrome, cellular senescence, blood cells, immunosenescence, inflammaging, senescence-associated secretory phenotype (SASP), clonal hematopoiesis, oxidative stress, mitochondrial dysfunction, metabolic inflammation

## Abstract

**Highlights:**

**What are the main findings?**

- Metabolic stress promotes senescence across hematopoietic stem cells and circulating immune-cell populations through interconnected oxidative, mitochondrial, DNA-damage, nutrient-sensing, autophagic, epigenetic, and inflammatory pathways.
- Senescent blood cells amplify inflammaging and immune dysfunction, providing a mechanistic link between metabolic syndrome, clonal hematopoiesis, and cardiometabolic disease.

**What are the implications of the main findings?**

- Composite blood-cell senescence signatures may complement conventional cardiometabolic risk assessment and treatment monitoring but require analytical standardization and prospective clinical validation.
- Senolytic, senomorphic, and inflammation-modulating strategies are promising, although longitudinal and interventional human studies are needed to establish causality, efficacy, and safety.

**Abstract:**

Metabolic syndrome (MetS) is a complex metabolic disorder characterized by central obesity, insulin resistance, dyslipidemia, hypertension, and chronic low-grade inflammation, all of which contribute to an increased risk of type 2 diabetes mellitus, cardiovascular disease, and premature mortality. Emerging evidence suggests that cellular senescence plays a central role in the pathophysiology of MetS by linking metabolic stress to chronic inflammation, immune dysfunction, as well as cell and tissue damage. Although senescence has traditionally been studied in tissue-resident cells, growing attention has focused on the role of blood-cell senescence in the initiation and progression of metabolic disease. This review summarizes current knowledge regarding the molecular and cellular mechanisms driving senescence in hematopoietic stem cells and circulating blood-cells (BCs) and how these mechanisms affect specific blood-cell populations leading to altered immune function, impaired tissue homeostasis, and persistent inflammatory activation. The link between clonal hematopoiesis to immunosenescence and cardiometabolic disease is also highlighted, providing additional insight into the complex interactions between hematopoietic aging and metabolic dysfunction.

## 1. Introduction

Metabolic syndrome (MetS) is a multifactorial disorder characterized by the coexistence of central obesity, insulin resistance, dyslipidemia, hypertension, and impaired glucose homeostasis, and is largely recognized as a major contributor to the global burden of chronic disease [1,2,3]. The prevalence of MetS has increased substantially over recent decades, following the rise in obesity and population aging. Individuals with MetS exhibit an increased risk of developing type 2 diabetes mellitus (T2DM), cardiovascular disease (CVD), metabolic dysfunction-associated steatotic liver disease (MASLD), and premature mortality [1,2,4]. Although the clinical manifestations and metabolic abnormalities associated with MetS have been extensively investigated, the biological mechanisms linking metabolic dysfunction to chronic inflammation, accelerated aging, and cardiometabolic complications remain incompletely understood.

Recent advances in aging biology have identified cellular senescence as a key modulator of the intersection of metabolism, inflammation, and age-related disease. Cellular senescence is a stress-induced cellular state characterized by irreversible cell-cycle arrest, metabolic reprogramming, resistance to apoptosis, and the acquisition of a senescence-associated secretory phenotype (SASP) [5,6]. Senescence can be induced by various stressors, including DNA damage (both within nucleus and circulating DNA fragments), telomere attrition, oxidative stress, mitochondrial dysfunction, and chronic metabolic overload [7,8]. Although initially beneficial by preventing the proliferation of damaged cells, the progressive accumulation of senescent cells contributes to tissue dysfunction and chronic inflammation through the sustained secretion of pro-inflammatory cytokines, chemokines, growth factors, and proteases [6,9]. A senescence-like phenotype (SLP) is a state where cells—often cancer cells—stop dividing and show physical traits of natural cellular aging after being exposed to stress like chemotherapy or radiation [10]. In the immune system, this age-associated phenomenon is termed immunosenescence and describes the dysregulation and remodeling of immune function resulting from cellular aging. Consequently, aging markedly impairs the functions of multiple cell types involved in both innate and adaptive immune responses, leading to alterations in lymphocyte subsets, reduced proliferative capacity, and persistent low-grade inflammation, among other changes. These age-related alterations increase the susceptibility of older individuals not only to infectious diseases but also to malignancies and autoimmune disorders [11].

Accumulating evidence suggests that metabolic disorders promote the premature development of cellular senescence. Obesity, insulin resistance, hyperglycemia, and dyslipidemia generate a cellular environment characterized by oxidative stress, mitochondrial impairment, and chronic inflammatory signaling, all of which favor senescence induction [12,13,14]. Recent human studies have demonstrated increased senescent-cell burden in individuals with obesity, T2DM, and MASLD, independent of chronological age, indicating that metabolic stress may accelerate biological aging processes [12,15]. Furthermore, senescent cells have been shown to contribute actively to metabolic dysfunction by amplifying inflammation and disrupting tissue homeostasis, suggesting the existence of a bidirectional relationship between metabolic disease and cellular senescence [13,16].

A hallmark of MetS is chronic low-grade systemic inflammation, commonly referred to as metaflammation [17]. Unlike acute inflammation, metaflammation is persistent and is largely driven by nutrient excess, adipose tissue dysfunction, and altered immune-cell activity [18]. The expansion of visceral adipose tissue promotes the recruitment and activation of immune cells, leading to increased production of inflammatory mediators that impair insulin signaling and metabolic regulation [8,19]. Increasing evidence indicates that cellular senescence and metaflammation are tightly interconnected processes. Metabolic stress promotes senescence induction, whereas senescent cells perpetuate inflammatory responses through SASP secretion, thereby creating a self-sustaining cycle that contributes to the development and progression of cardiometabolic disease [13,16,20].

While early studies primarily focused on senescence within metabolic tissues such as adipose tissue, liver, skeletal muscle, and the vasculature, recent evidence has highlighted the importance of circulating blood cells (BCs) as both targets and mediators of metabolic dysfunction [19,20,21]. Senescence-associated alterations have been identified in several immune-cell populations, including T lymphocytes, B lymphocytes, monocytes, macrophages, and natural killer cells in individuals with obesity, insulin resistance, and T2DM [22,23,24,25]. These alterations include telomere shortening, mitochondrial dysfunction, impaired proliferative capacity, modified cellular metabolism, and increased expression of senescence-associated markers such as p16INK4a and p21 [23,24]. Importantly, such changes closely resemble the phenotype of immunosenescence, a process characterized by age-associated deterioration of immune function and chronic low-grade inflammation [26].

Among circulating immune cells, T lymphocytes appear to play a pivot role in the pathogenesis of metabolic disease. Recent studies have demonstrated that senescent T-cell populations accumulate in obesity and T2DM and are associated with insulin resistance, impaired glucose homeostasis, and increased systemic inflammation [22,24,27]. These cells exhibit altered metabolic programming, reduced immune competence, and enhanced secretion of inflammatory mediators, suggesting that immune-cell senescence may actively contribute to disease progression rather than simply reflect metabolic dysfunction [22,27]. Moreover, emerging evidence indicates that obesity can induce premature immunosenescent phenotypes, supporting the concept that MetS accelerates biological aging processes through immune-mediated mechanisms [20,24].

Beyond their pathogenic significance, senescent blood-cells are increasingly being investigated as accessible biomarkers of biological aging and metabolic risk [28]. Circulating immune cells provide a readily available source for the assessment of senescence-associated molecular signatures, including telomere attrition, p16INK4a expression, mitochondrial dysfunction, epigenetic alterations, and inflammatory cytokine profiles [28,29]. In parallel, the development of senotherapeutic approaches, including senolytics and senomorphics, has generated considerable interest in targeting senescent cells as a novel strategy for the prevention and treatment of cardiometabolic disease. Experimental and early clinical studies suggest that reducing senescent-cell burden or modulating SASP activity may alleviate inflammation and improve metabolic homeostasis [30,31].

Therefore, in this review we examine the current evidence regarding cellular senescence in circulating blood and immune cells within the context of MetS. Emphasis is placed on the molecular mechanisms linking metabolic stress to senescence induction, the contribution of senescent immune cells to chronic inflammation and metabolic dysregulation, and their utility as biomarkers of biological aging. Most mechanistic evidence linking blood-cell senescence to metabolic dysfunction derives from in vitro experiments and animal models, which allow for pathway-specific interrogation but may not fully reproduce the cellular heterogeneity, exposure history, comorbidities, and treatment context of human MetS. Conversely, human studies are predominantly cross-sectional or observational and identify correlations between senescence-associated markers and metabolic outcomes without establishing temporal direction or excluding reverse causation and residual confounding. Nevertheless, by integrating recent findings from the fields of geroscience, immunology, and metabolic medicine, this review proposes that blood-cell senescence represents a critical mechanistic nexus connecting MetS, immunosenescence, inflammaging, and cardiometabolic disease.

## 2. Cellular Senescence in Blood Cells: Molecular Pathways Linking Metabolic Syndrome to Blood Cells Aging

Accumulating evidence suggests that MetS accelerates cellular senescence throughout the hematopoietic and immune systems, thereby contributing to chronic inflammation, immune dysfunction, and increased cardiovascular risk. Rather than representing isolated phenomena, metabolic dysregulation and cellular senescence participate in a bidirectional relationship in which obesity, insulin resistance, dyslipidemia, and chronic low-grade inflammation promote senescence, while senescent cells further exacerbate metabolic abnormalities through the secretion of pro-inflammatory mediators. At the molecular level, this interaction is mediated by a complex network of signaling pathways involving oxidative stress, DNA damage responses, mitochondrial dysfunction, inflammatory signaling, altered nutrient sensing, epigenetic remodeling, and impaired autophagy [21,32,33,34].

### 2.1. Oxidative Stress and Mitochondrial Dysfunction

**Figure 1:**
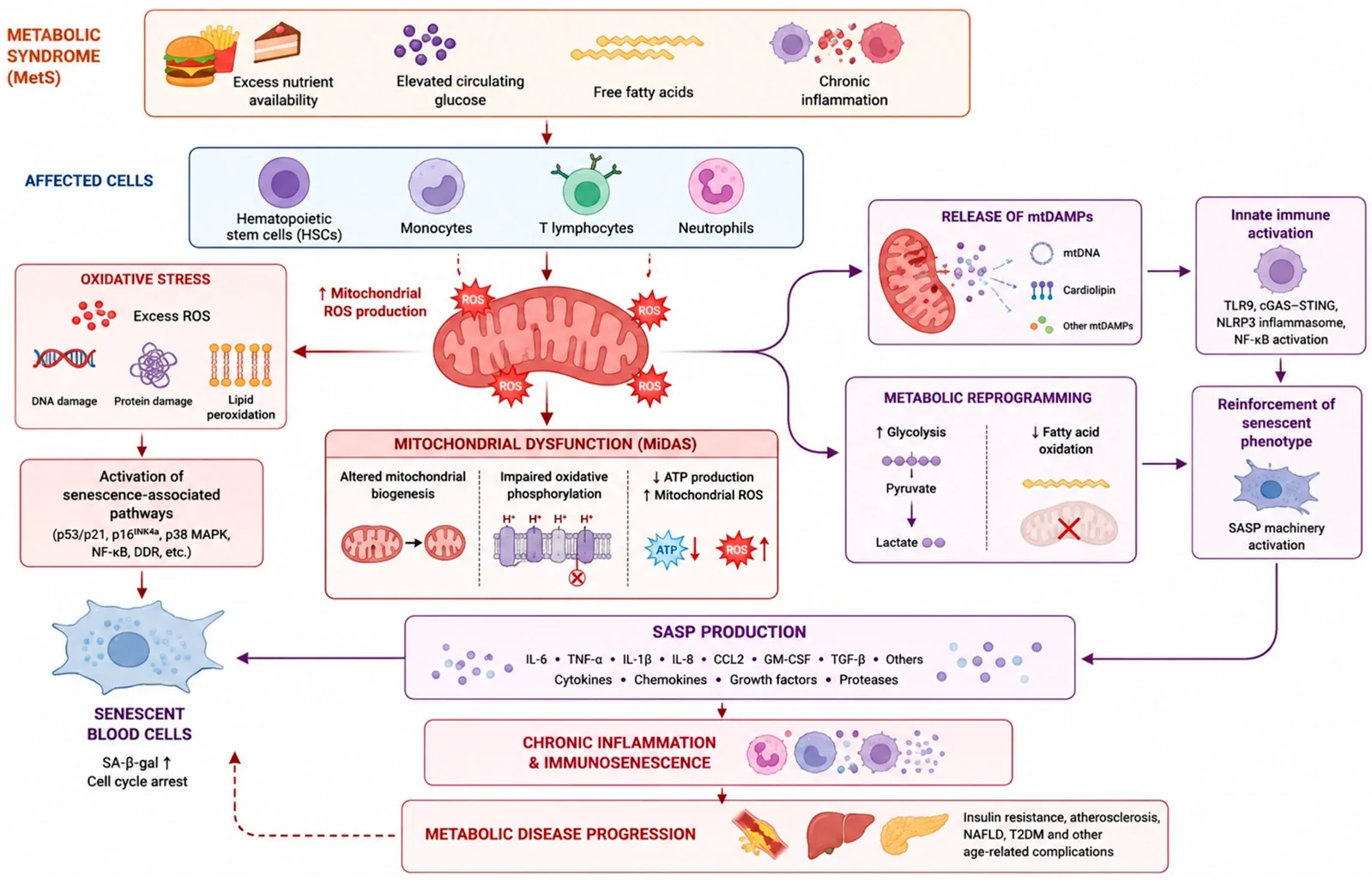
Oxidative stress and mitochondrial dysfunction of blood cells in metabolic syndrome. Abbreviations: ATP, adenosine triphosphate; cGAS–STING, cyclic GMP–AMP synthase–stimulator of interferon genes; CCL2, C–C motif chemokine ligand 2; DDR, DNA damage response; GM-CSF, granulocyte–macrophage colony-stimulating factor; HSCs, hematopoietic stem cells; IL, interleukin; IL-1β, interleukin-1 beta; IL-6, interleukin-6; IL-8, interleukin-8; MAPK, mitogen-activated protein kinase; MetS, metabolic syndrome; MiDAS, mitochondrial dysfunction-associated senescence; mtDAMPs, mitochondrial damage-associated molecular patterns; mtDNA, mitochondrial DNA; NAFLD, non-alcoholic fatty liver disease; NF-κB, nuclear factor kappa B; NLRP3, NOD-, LRR- and pyrin domain-containing protein 3; ROS, reactive oxygen species; SA-β-gal, senescence-associated beta-galactosidase; SASP, senescence-associated secretory phenotype; TGF-β, transforming growth factor beta; TLR9, Toll-like receptor 9; TNF-α, tumor necrosis factor alpha; T2DM, type 2 diabetes mellitus.

Oxidative stress represents one of the primary drivers of blood-cell senescence in MetS (Figure 1). Excess nutrient availability, elevated circulating glucose concentrations, free fatty acids, and chronic inflammation enhance mitochondrial production of reactive oxygen species (ROS) in hematopoietic stem cells (HSCs), monocytes, lymphocytes, and neutrophils [21]. While physiological ROS levels participate in intracellular signaling, excessive ROS production causes oxidative damage to DNA, proteins, and lipids, modulating senescence-associated pathways.

Mitochondria play a central role in this process. Senescent immune cells exhibit altered mitochondrial biogenesis, impaired oxidative phosphorylation, reduced ATP production, and increased mitochondrial ROS generation [35]. Damaged mitochondria release mitochondrial DNA (mtDNA), cardiolipin, and other mitochondrial danger-associated molecular patterns (mtDAMPs) that activate innate immune signaling pathways and perpetuate inflammatory responses. This phenomenon, known as mitochondrial dysfunction-associated senescence (MiDAS), is increasingly recognized as a major contributor to immunosenescence and metabolic disease progression [35]. In addition, during innate immune activation, mitochondrial dysfunction promotes the release of mtDAMPs, including mtDNA and cardiolipin, into intracellular compartments or the extracellular environment. Endosomal TLR9 can recognize CpG-rich mtDNA, whereas cytosolic mtDNA activates the cGAS–STING pathway. Mitochondrial stress signals, including oxidized mtDNA, mitochondrial ROS and cardiolipin, can additionally promote activation of the NLRP3 inflammasome. These pathways converge on inflammatory signaling, including NF-κB activation and inflammasome-dependent maturation of IL-1β, thereby increasing the production of cytokines and other SASP components. Persistent activation of these innate immune pathways reinforces the senescent phenotype and contributes to chronic inflammation, immunosenescence and metabolic-disease progression [35].

Furthermore, mitochondrial dysfunction alters cellular metabolism by promoting glycolytic reprogramming and disrupting fatty acid oxidation, processes that are particularly relevant in metabolically active immune cells such as monocytes and T lymphocytes. These alterations reinforce the senescent phenotype and contribute to SASP production [19].

### 2.2. DNA Damage Response and Cell-Cycle Arrest Pathways

**Figure 2:**
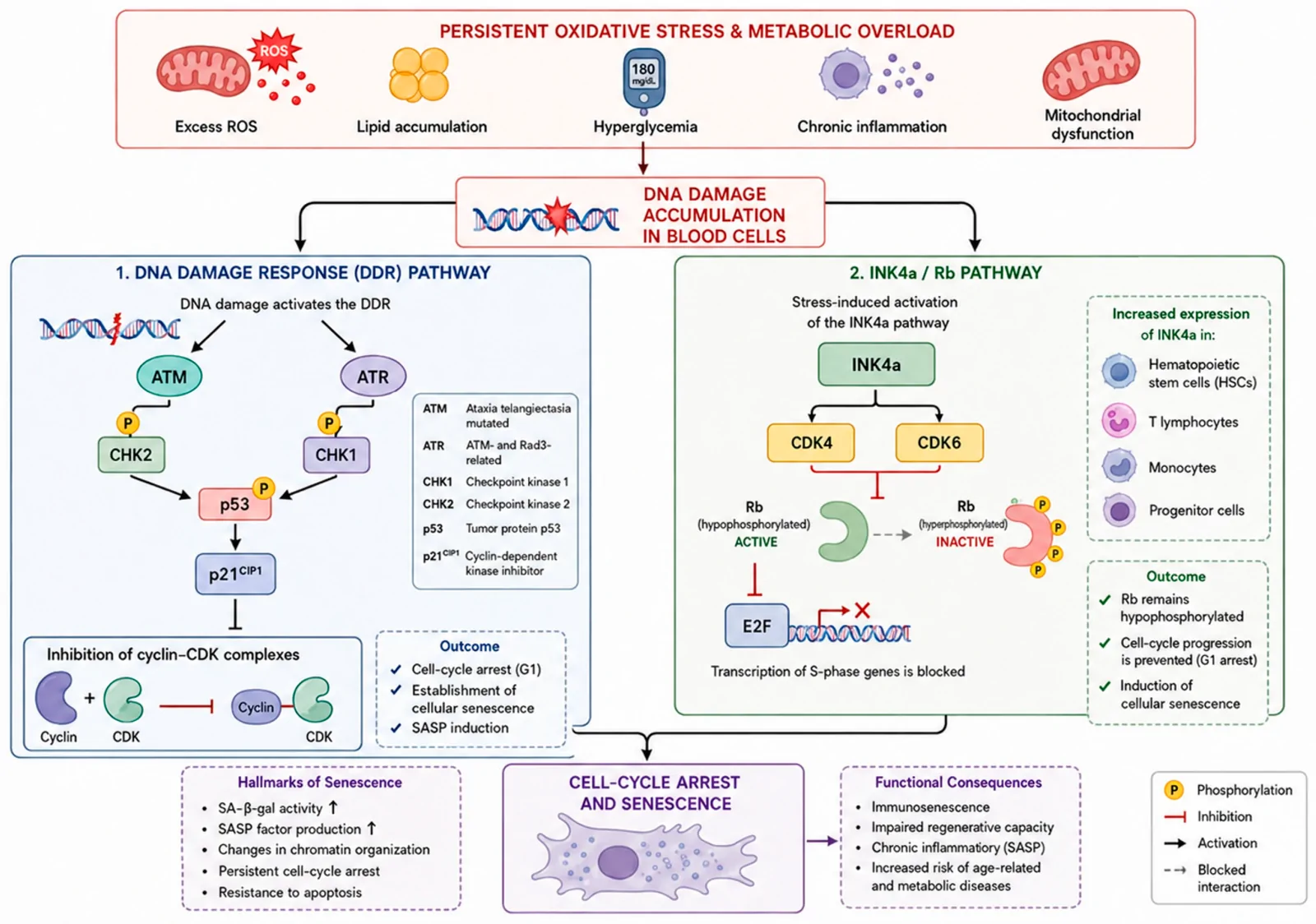
DNA damage related mechanisms of blood cells in metabolic syndrome. Abbreviations: ATM, ataxia-telangiectasia mutated; ATR, ATM- and Rad3-related; CDK, cyclin-dependent kinase; CDK4, cyclin-dependent kinase 4; CDK6, cyclin-dependent kinase 6; CHK1, checkpoint kinase 1; CHK2, checkpoint kinase 2; DDR, DNA damage response; DNA, deoxyribonucleic acid; E2F, E2F transcription factor; G1, gap 1 phase of the cell cycle; HSCs, hematopoietic stem cells; INK4a, inhibitor of cyclin-dependent kinase 4a; MAPK, mitogen-activated protein kinase; p21CIP1, p21 cyclin-dependent kinase inhibitor 1; p53, tumor protein p53; Rb, retinoblastoma protein; ROS, reactive oxygen species; SA-β-gal, senescence-associated beta-galactosidase; SASP, senescence-associated secretory phenotype.

Persistent oxidative stress and metabolic overload induce DNA damage accumulation in BCs (Figure 2). Damaged DNA activates the DNA damage response (DDR), a highly conserved signaling network coordinated by the phosphatidylinositol 3-kinase-related kinases ATM (ataxia telangiectasia mutated) and ATR (ATM- and Rad3-related). Activation of ATM and ATR leads to phosphorylation of downstream effectors such as CHK1 (Checkpoint kinase 1), CHK2 (Checkpoint kinase 2), and p53, ultimately inducing expression of the cyclin-dependent kinase inhibitor p21CIP1 [34]. The resulting inhibition of cyclin-CDK (Cyclin-Dependent Kinase) complexes produces cell-cycle arrest and facilitates the establishment of cellular senescence.

In parallel, stress-induced activation of the INK4a/Rb pathway represents a second major mechanism of senescence induction, since INK4a (inhibitor of CDK4) and Rb (retinoblastoma protein) are critical tumor suppressor proteins that control the cell cycle, while INK4a acts as a major biomarker for cellular aging. Increased expression of INK4a inhibits CDK4 and CDK6 activity, maintaining the Rb protein in its hypophosphorylated state and thereby preventing cell-cycle progression [36]. Increased expression of INK4a has been consistently reported in aged hematopoietic stem cells, T lymphocytes, monocytes, and progenitor populations, making it one of the most widely used biomarkers of cellular senescence in human BCs [36].

### 2.3. Telomere Attrition and Replicative Senescence

Telomere shortening constitutes another important mechanism contributing to BCs senescence. Telomeres are repetitive DNA sequences located at chromosome ends that progressively shorten during successive cell division rounds. Critically short telomeres are recognized as DNA damage, activating ATM-dependent signaling and triggering p53-mediated senescence programs [37].

Several studies have demonstrated accelerated telomere shortening in leukocytes from individuals with obesity, insulin resistance, type 2 diabetes, and MetS [38,39]. Chronic inflammation and oxidative stress appear to accelerate telomere erosion beyond that expected from chronological aging alone. Shortened leukocyte telomere length has been associated with increased cardiovascular risk, impaired immune function, and higher mortality in metabolic disease cohorts.

### 2.4. NF-κB Signaling and the Senescence-Associated Secretory Phenotype

A defining characteristic of senescent cells is the acquisition of SASP, a complex pro-inflammatory secretome that includes cytokines, chemokines, growth factors, matrix-remodeling enzymes, and extracellular vesicles [38,39,40].

**Figure 3:**
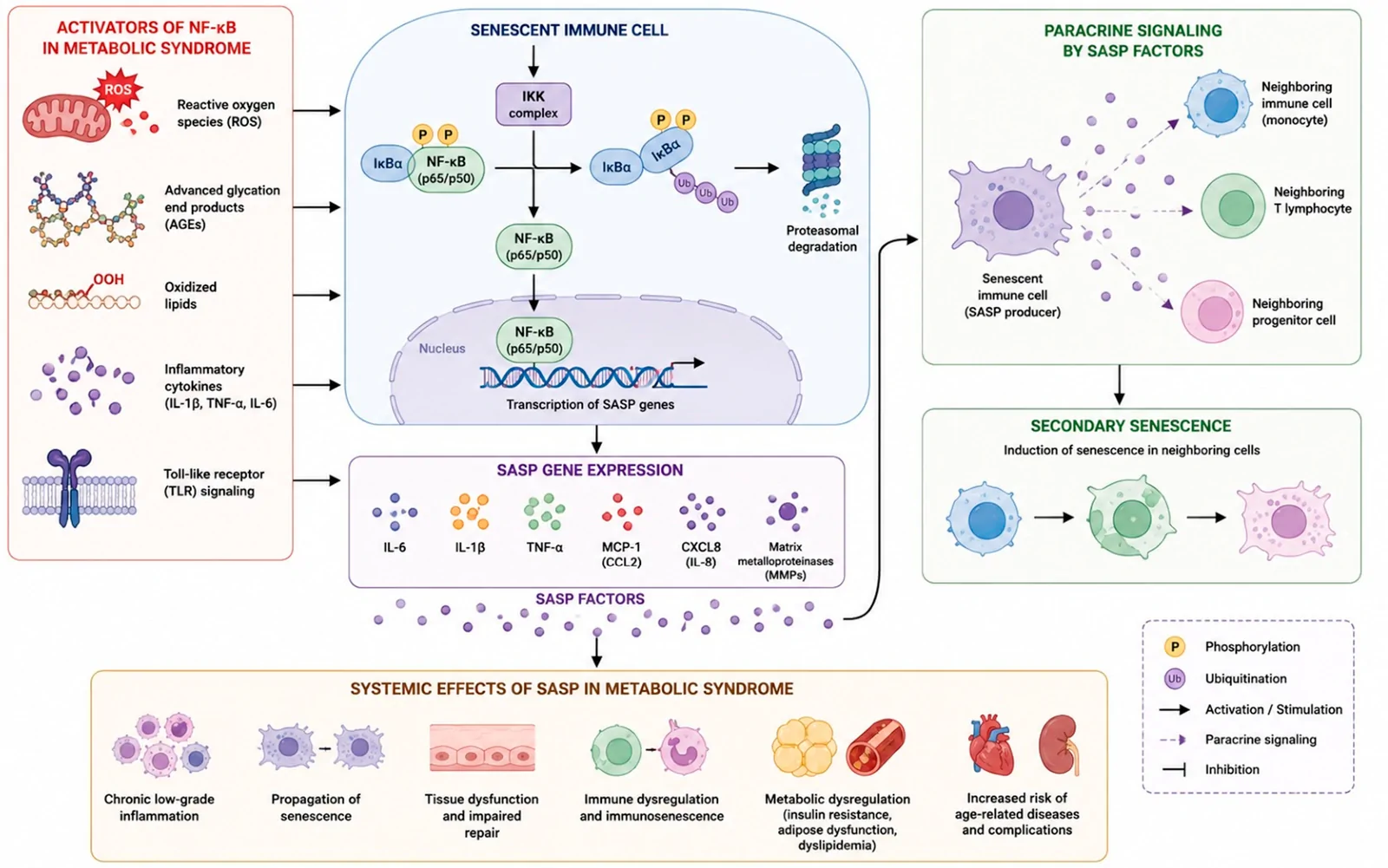
NF-κB Signaling and SASP of blood cells in metabolic syndrome. Abbreviations: AGEs, advanced glycation end products; CCL2, C–C motif chemokine ligand 2; CXCL8, C–X–C motif chemokine ligand 8; IKK, inhibitor of nuclear factor kappa B kinase; IκBα, inhibitor of nuclear factor kappa B alpha; IL-1β, interleukin-1 beta; IL-6, interleukin-6; IL-8, interleukin-8; MCP-1, monocyte chemoattractant protein 1; MMPs, matrix metalloproteinases; NF-κB, nuclear factor kappa B; p50, nuclear factor kappa B subunit 1; p65, RelA proto-oncogene/NF-κB subunit; ROS, reactive oxygen species; SASP, senescence-associated secretory phenotype; TLR, Toll-like receptor; TNF-α, tumor necrosis factor alpha; P, phosphorylation; Ub, ubiquitination.

The transcription factor NF-κB serves as a master regulator of SASP expression (Figure 3). In MetS, NF-κB is activated by ROS, advanced glycation end products (AGEs), oxidized lipids, inflammatory cytokines, and Toll-like receptor signaling [19]. Activated NF-κB promotes transcription of IL-6 (Interleukin-6), IL-1β (Interleukin-1 beta), TNF-α (Tumor Necrosis Factor alpha), MCP-1(Monocyte Chemoattractant Protein-1), IL-8 (Interleukin-8), and matrix metalloproteinases, generating a chronic inflammatory environment [41].

Importantly, SASP factors act not only locally but also systemically. Through paracrine signaling, SASP components induce senescence in neighboring cells, a process termed “secondary senescence.” Consequently, a relatively small number of senescent immune cells may exert disproportionate effects on tissue homeostasis and metabolic regulation [34].

### 2.5. NLRP3 Inflammasome Activation

**Figure 4:**
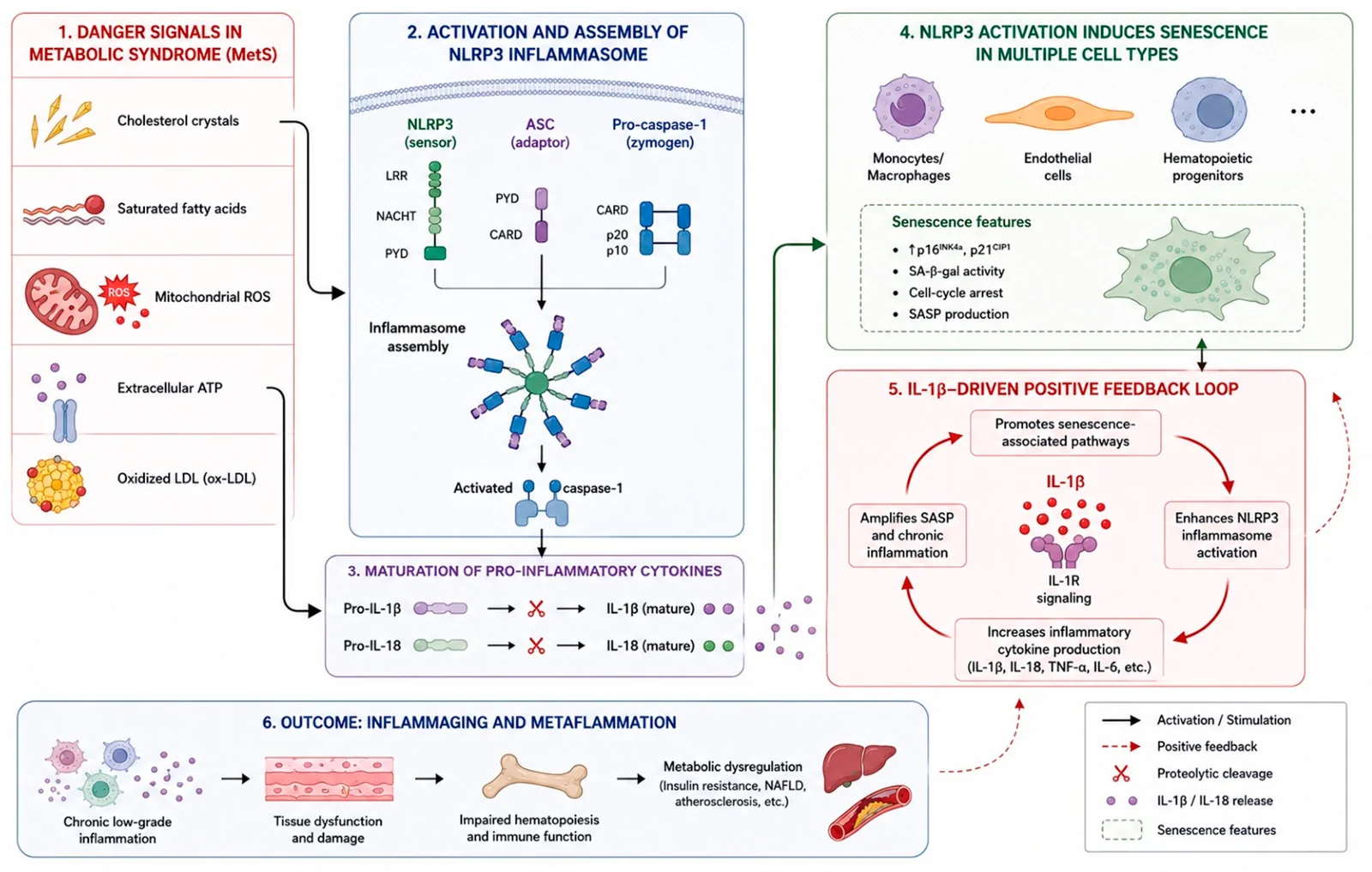
NLRP3 inflammasome activation of blood cells in metabolic syndrome. Abbreviations: ASC, apoptosis-associated speck-like protein containing a caspase activation and recruitment domain; ATP, adenosine triphosphate; CARD, caspase activation and recruitment domain; IL-1β, interleukin-1 beta; IL-1R, interleukin-1 receptor; IL-6, interleukin-6; IL-18, interleukin-18; LDL, low-density lipoprotein; LRR, leucine-rich repeat; MetS, metabolic syndrome; NACHT, NAIP, CIITA, HET-E and TP1 domain; NAFLD, non-alcoholic fatty liver disease; NLRP3, NOD-, LRR- and pyrin domain-containing protein 3; ox-LDL, oxidized low-density lipoprotein; p16INK4a, p16 inhibitor of cyclin-dependent kinase 4a; p21CIP1, p21 cyclin-dependent kinase inhibitor 1; p20 and p10, the catalytically active cleavage subunits of caspase-1; PYD, pyrin domain; ROS, reactive oxygen species; SA-β-gal, senescence-associated beta-galactosidase; SASP, senescence-associated secretory phenotype; TNF-α, tumor necrosis factor alpha.

The NLRP3 inflammasome has emerged as a critical molecular link between metabolic stress and cellular senescence (Figure 4). NLRP3 is activated by numerous danger signals present in MetS, including cholesterol crystals, saturated fatty acids, mitochondrial ROS, extracellular ATP, and oxidized LDL (Low-Density Lipoprotein) [42].

Activation of the NLRP3 inflammasome leads to assembly of a multiprotein complex containing NLRP3(NOD-, LRR- and pyrin domain-containing protein 3), ASC (Apoptosis-associated speck-like protein containing a CARD), and caspase-1. Activated caspase-1 subsequently cleaves pro-IL-1β and pro-IL-18 into their mature inflammatory forms, amplifying inflammatory signaling [43].

Recent evidence indicates that NLRP3 activation is closely associated with senescence induction in monocytes, macrophages, endothelial cells, and hematopoietic progenitors. Furthermore, IL-1β itself can promote senescence-associated pathways, establishing a positive feedback loop that reinforces both inflammaging and metaflammation [21].

### 2.6. mTOR, AMPK, and Nutrient-Sensing Pathways

Cellular senescence is strongly influenced by nutrient-sensing pathways that regulate metabolism, growth, and stress responses. Among these, mechanistic target of rapamycin (mTOR) represents a central regulator of senescence development.

MetS is characterized by chronic activation of mTOR signaling due to nutrient excess, hyperinsulinemia, and increased IGF-1 signaling [44]. Persistent mTOR activation promotes protein synthesis, mitochondrial dysfunction, SASP production, and cellular hypertrophy, all hallmarks of senescent cells.

Conversely, AMP-activated protein kinase (AMPK), a key energy sensor, exerts anti-senescent effects by suppressing mTOR activity, promoting autophagy, enhancing mitochondrial quality control, and reducing oxidative stress [45]. In obesity and insulin resistance, AMPK activity is often diminished, favoring senescence progression.

Dysregulation of the mTOR-AMPK axis therefore represents a major molecular mechanism linking metabolic overload to immune-cell aging.

### 2.7. Autophagy Impairment and Loss of Proteostasis

Autophagy is an essential cellular housekeeping process responsible for degradation of damaged proteins, dysfunctional mitochondria, and intracellular debris. Efficient autophagic activity is crucial for maintaining immune-cell homeostasis and preventing senescence [46].

In MetS, chronic nutrient excess suppresses autophagy through mTOR activation, resulting in accumulation of dysfunctional organelles and increased oxidative stress. Impaired autophagy has been documented in monocytes, lymphocytes, HSCs, and adipose tissue macrophages from obese individuals [33].

Defective autophagy contributes directly to senescence by enhancing DNA damage accumulation, mitochondrial dysfunction, inflammasome activation, and SASP production, thereby reinforcing multiple senescence-promoting pathways simultaneously.

### 2.8. Epigenetic Remodeling, Trained Immunity, and cGAS-STING Signaling

Recent studies have highlighted the importance of epigenetic remodeling in blood-cell senescence. Chronic metabolic stress induces long-lasting changes in DNA methylation, histone modifications, and chromatin organization that alter gene expression patterns and promote inflammatory phenotypes [47,48].

These changes contribute to trained immunity, a form of innate immune memory characterized by sustained inflammatory responsiveness of monocytes and myeloid progenitors. Hyperglycemia and dyslipidemia induce metabolic and epigenetic reprogramming that persists even after normalization of metabolic parameters, suggesting a molecular basis for the phenomenon of “metabolic memory” [47].

In parallel, cytoplasmic chromatin fragments and damaged mitochondrial DNA activate cyclic GMP-AMP synthase (cGAS), leading to stimulation of the STING pathway, a core part of the human innate immune system that detects foreign or damaged DNA inside a cell’s cytoplasm and triggers a protective immune response, and induction of type I interferon responses [49]. Emerging evidence indicates that cGAS-STING signaling is a major driver of SASP production, chronic inflammation, and senescence propagation in aging and metabolic disease.

Collectively, these interconnected molecular pathways establish a self-reinforcing network linking metabolic dysfunction to hematopoietic aging, immune-cell senescence, chronic inflammation, and cardiometabolic disease.

## 3. Clonal Hematopoiesis and MetS

Clonal hematopoiesis (CH) has emerged as an important link between hematopoietic aging, chronic inflammation, and cardiometabolic disease. CH is characterized by the age-associated expansion of hematopoietic stem and progenitor cell (HSPC) clones carrying acquired somatic mutations that confer a competitive advantage over normal stem cells. When such mutations are detected in the absence of overt hematological malignancy and with a variant allele frequency of at least 2%, the condition is referred to as clonal hematopoiesis of indeterminate potential (CHIP) [50].

The prevalence of CHIP increases markedly with age, affecting less than 1% of individuals younger than 40 years but more than 10–20% of individuals over 70 years of age [51,52]. The most frequently mutated genes include *DNMT3A*, *TET2*, *ASXL1*, *JAK2*, and *TP53*, all of which regulate epigenetic programming, DNA repair, stem-cell self-renewal, or inflammatory signaling. Although CHIP was initially recognized as a precursor state for hematologic malignancies, accumulating evidence demonstrates its broader role in age-related disorders, particularly cardiovascular and metabolic diseases [53].

Several mechanisms connect CHIP with cellular senescence and MetS. Aging hematopoietic stem cells are continuously exposed to oxidative stress, chronic inflammation, DNA damage, and telomere attrition, conditions that favor both senescence and the selection of mutant clones. Senescent HSCs exhibit impaired regenerative capacity and altered differentiation patterns, creating a hematopoietic environment in which mutant clones may gain a selective advantage. In turn, CHIP-associated mutations can exacerbate inflammatory responses and further promote tissue dysfunction, establishing a vicious cycle between stem-cell aging, clonal expansion, and chronic inflammation.

Among CHIP-associated mutations, *TET2* deficiency has been extensively studied in the context of cardiometabolic disease. Experimental studies have shown that loss of *TET2* function in hematopoietic cells enhances activation of the NLRP3 inflammasome and increases the production of pro-inflammatory cytokines such as interleukin-1β (IL-1β) and interleukin-6 (IL-6) [54]. Similarly, mutations in *DNMT3A* have been associated with altered immune-cell differentiation and increased inflammatory signaling. These inflammatory pathways overlap substantially with the mechanisms driving SASP production, suggesting that CHIP and cellular senescence may reinforce each other through shared molecular networks.

Emerging evidence indicates that CHIP is more prevalent among individuals with obesity, insulin resistance, type 2 diabetes mellitus, and other components of MetS. Chronic metabolic stress may promote the expansion of mutant hematopoietic clones through increased oxidative damage and inflammatory signaling, while CHIP-derived immune cells may contribute to worsening metabolic dysfunction. In animal models, *TET2*-mutant macrophages accelerate adipose tissue inflammation, insulin resistance, and atherosclerotic plaque development, highlighting the bidirectional relationship between clonal hematopoiesis and metabolic disease [54,55].

The clinical significance of CHIP extends beyond hematopoiesis. Large population studies have demonstrated that CHIP is associated with a substantially increased risk of cardiovascular disease, myocardial infarction, stroke, and all-cause mortality, independent of traditional cardiovascular risk factors [56]. Given that MetS itself is a major driver of cardiovascular morbidity, the coexistence of CHIP and metabolic dysfunction may amplify disease progression through persistent inflammation, endothelial dysfunction, and immune dysregulation.

Recent findings suggest that CHIP may also contribute directly to immunosenescence. Mutated hematopoietic clones generate myeloid cells with pro-inflammatory phenotypes that resemble senescent immune cells, including enhanced cytokine secretion and dysregulated innate immune responses. These alterations may perpetuate inflammaging and promote the accumulation of senescent cells in peripheral tissues. Consequently, CHIP is increasingly viewed not only as a marker of hematopoietic aging but also as an active participant in the pathophysiology of senescence-related disorders [56].

Taken together, clonal hematopoiesis represents a critical intersection between aging, blood-cell senescence, chronic inflammation, and metabolic disease. Future studies are needed to clarify the causal relationships among CHIP, immunosenescence, and MetS and to determine whether interventions targeting senescent cells or inflammatory pathways can mitigate the adverse clinical consequences associated with clonal hematopoiesis.

## 4. Senescence Molecular Pathways of Specific BCs Sub-Populations in MetS

**Figure 5:**
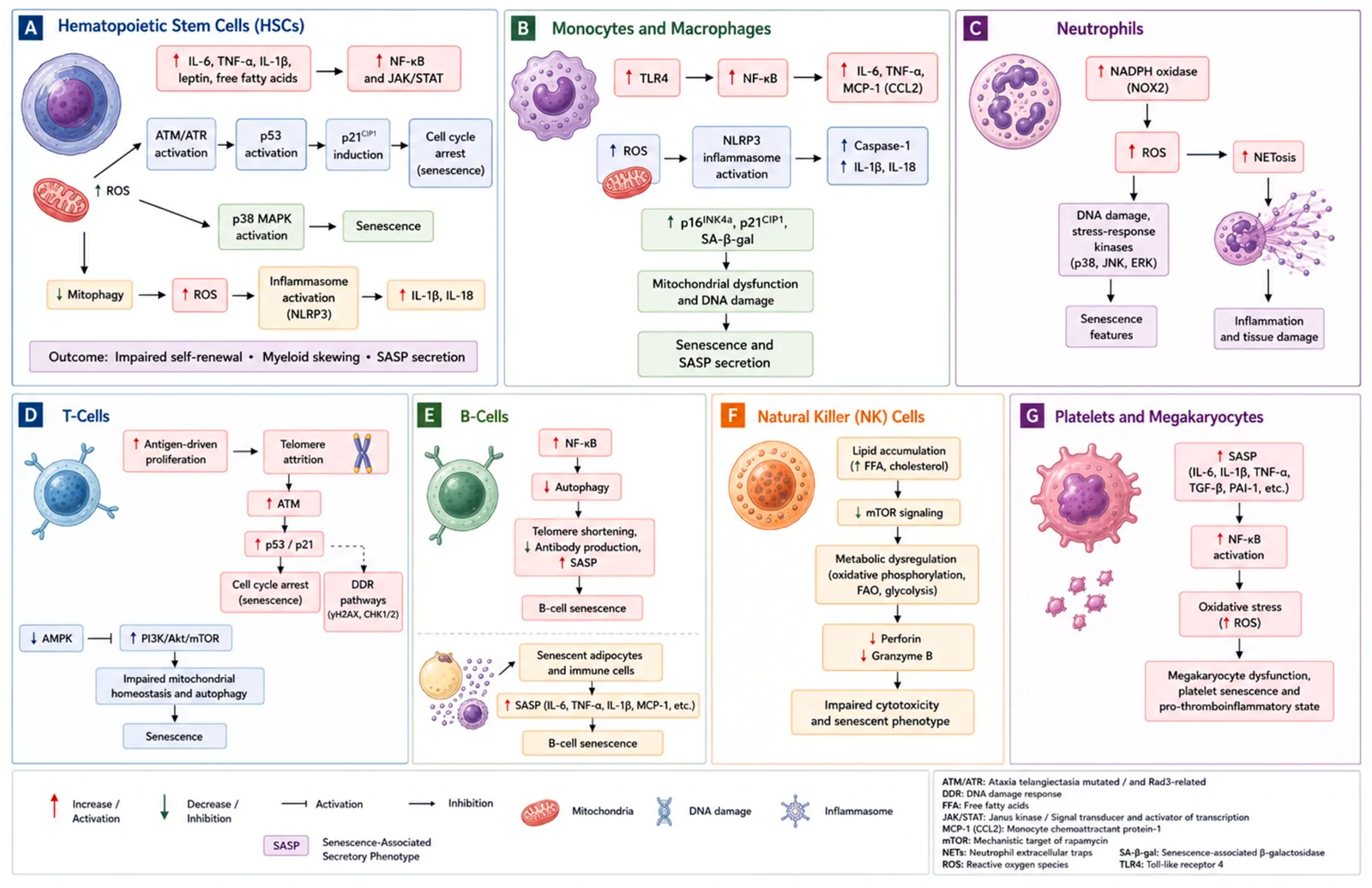
Senescence molecular pathways of specific blood cells sub-populations in metabolic syndrome. (**A**) Hematopoietic stem cells (HSCs); (**B**) Monocytes and macrophages; (**C**) Neutrophils; (**D**) T cells; (**E**): B cells; (**F**): Natural killer (NK) cells; (**G**): Platelets and megakaryocytes. Abbreviations: Akt, protein kinase B; AMPK, AMP-activated protein kinase; ATM, ataxia-telangiectasia mutated; ATR, ATM- and Rad3-related; CCL2, C–C motif chemokine ligand 2; CHK1/2, checkpoint kinases 1 and 2; DDR, DNA damage response; DNA, deoxyribonucleic acid; ERK, extracellular signal-regulated kinase; FAO, fatty acid oxidation; FFA, free fatty acids; HSCs, hematopoietic stem cells; IL-1β, interleukin-1 beta; IL-6, interleukin-6; IL-8, interleukin-8; IL-18, interleukin-18; JAK, Janus kinase; JNK, c-Jun N-terminal kinase; MAPK, mitogen-activated protein kinase; MCP-1, monocyte chemoattractant protein 1; mTOR, mechanistic target of rapamycin; NADPH, reduced nicotinamide adenine dinucleotide phosphate; NETs, neutrophil extracellular traps; NETosis, neutrophil extracellular trap formation; NF-κB, nuclear factor kappa B; NK, natural killer; NLRP3, NOD-, LRR- and pyrin domain-containing protein 3; NOX2, NADPH oxidase 2; p16INK4a, p16 inhibitor of cyclin-dependent kinase 4a; p21CIP1, p21 cyclin-dependent kinase inhibitor 1; p38 MAPK, p38 mitogen-activated protein kinase; p53, tumor protein p53; PAI-1, plasminogen activator inhibitor 1; PI3K, phosphoinositide 3-kinase; ROS, reactive oxygen species; SA-β-gal, senescence-associated beta-galactosidase; SASP, senescence-associated secretory phenotype; STAT, signal transducer and activator of transcription; TGF-β, transforming growth factor beta; TLR4, Toll-like receptor 4; TNF-α, tumor necrosis factor alpha; γH2AX, phosphorylated histone H2AX.

The molecular mechanisms discussed above collectively contribute to the development of cellular senescence within the hematopoietic system. However, the consequences of these processes are not uniform across all BCs types. Different hematopoietic and immune cell populations exhibit distinct susceptibilities to senescence and develop characteristic functional alterations that contribute to the pathophysiology of MetS (Figure 5).

### 4.1. Hematopoietic Stem Cell Senescence

Hematopoietic stem cells (HSCs) maintain lifelong hematopoiesis through their unique capacity for self-renewal and differentiation into all mature blood-cell lineages. Growing evidence suggests that MetS accelerates HSC aging through mechanisms involving oxidative stress, chronic inflammation, mitochondrial dysfunction, and epigenetic remodeling (Figure 5A) [57,58,59].

In obesity and insulin resistance, increased systemic concentrations of IL-6, TNF-α, IL-1β, leptin, and free fatty acids induce persistent activation of NF-κB and JAK/STAT signaling within the bone marrow niche [58]. Simultaneously, elevated ROS levels activate ATM/ATR-mediated DNA damage responses, resulting in increased p53 and p21 expression and subsequent cell-cycle arrest [60]. Chronic oxidative stress also promotes activation of p38 MAPK (mitogen-activated protein kinase) signaling, a key regulator of stem-cell exhaustion and senescence.

**Figure 6:**
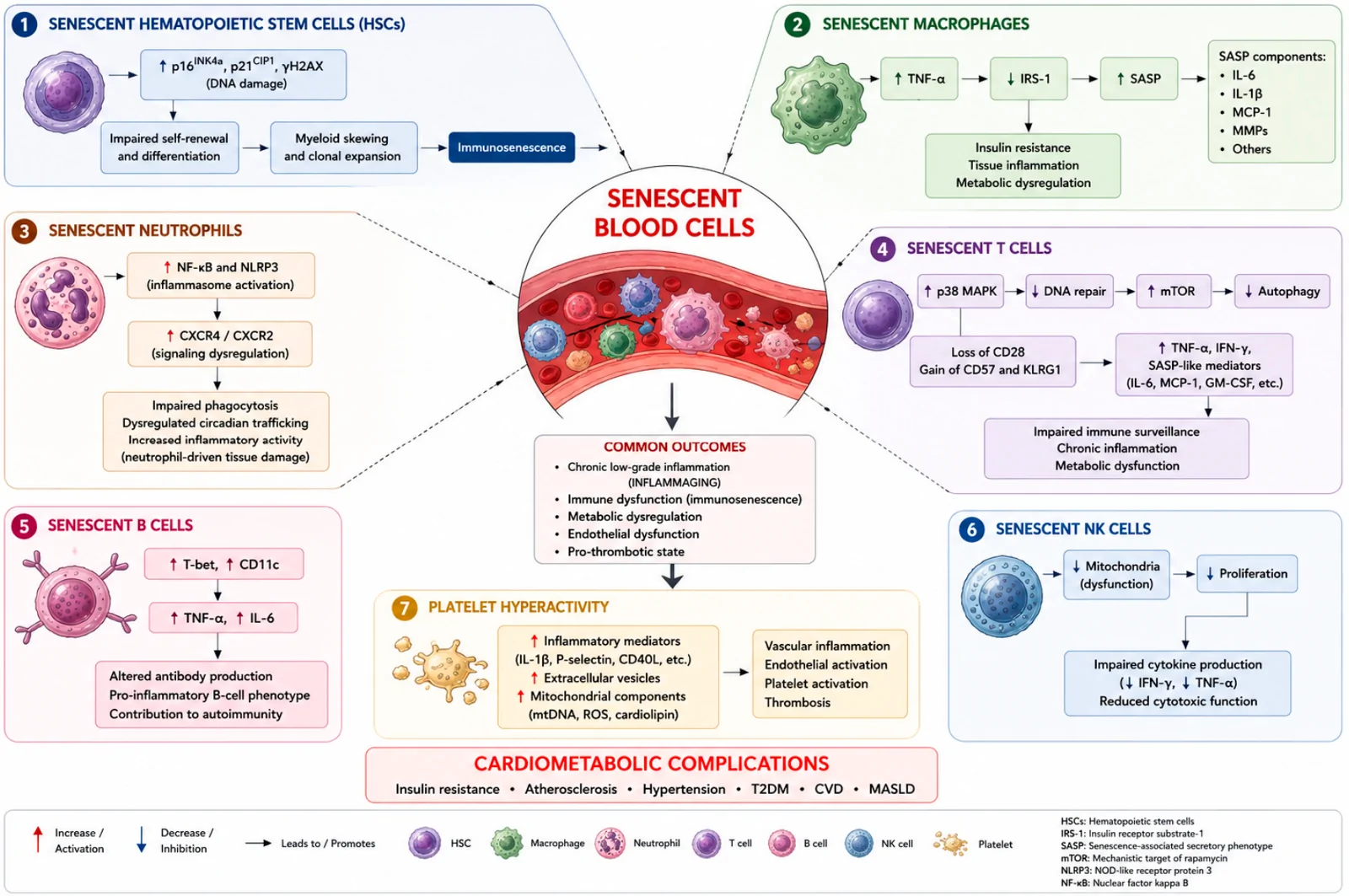
Senescent blood cells phenotype in metabolic syndrome. Abbreviations: CD11c, cluster of differentiation 11c/integrin alpha X; CD28, cluster of differentiation 28; CD40L, CD40 ligand; CD57, cluster of differentiation 57; CVD, cardiovascular disease; CXCR2, C–X–C chemokine receptor 2; CXCR4, C–X–C chemokine receptor 4; DNA, deoxyribonucleic acid; GM-CSF, granulocyte–macrophage colony-stimulating factor; HSCs, hematopoietic stem cells; IFN-γ, interferon gamma; IL-1β, interleukin-1 beta; IL-6, interleukin-6; IRS-1, insulin receptor substrate 1; KLRG1, killer cell lectin-like receptor subfamily G member 1; MAPK, mitogen-activated protein kinase; MASLD, metabolic dysfunction-associated steatotic liver disease; MCP-1, monocyte chemoattractant protein 1; MMPs, matrix metalloproteinases; mtDNA, mitochondrial DNA; mTOR, mechanistic target of rapamycin; NF-κB, nuclear factor kappa B; NK, natural killer; NLRP3, NOD-, LRR- and pyrin domain-containing protein 3; p16INK4a, cyclin-dependent kinase inhibitor 2A/p16 inhibitor of cyclin-dependent kinase 4a; p21CIP1, cyclin-dependent kinase inhibitor 1A; p38 MAPK, p38 mitogen-activated protein kinase; ROS, reactive oxygen species; SA-β-gal, senescence-associated beta-galactosidase; SASP, senescence-associated secretory phenotype; T-bet, T-box transcription factor TBX21; T2DM, type 2 diabetes mellitus; TNF-α, tumor necrosis factor alpha; γH2AX, phosphorylated histone H2AX.

Senescent HSCs exhibit increased expression of p16INK4a, p21CIP1, γH2AX, and senescence-associated inflammatory mediators [58,61,62]. Functionally, these alterations result in impaired self-renewal, reduced regenerative potential, and skewing toward myeloid differentiation at the expense of lymphopoiesis. This phenomenon contributes to immunosenescence by reducing adaptive immune-cell production while increasing the generation of inflammatory myeloid cells (Figure 6).

Mitochondrial dysfunction further accelerates HSC senescence. Reduced mitophagy and impaired mitochondrial quality control lead to accumulation of damaged mitochondria, increased ROS generation, and activation of inflammasome pathways [63]. Recent studies suggest that age-related clonal hematopoiesis may represent an advanced manifestation of HSC aging, linking senescence-associated hematopoietic dysfunction to atherosclerosis, cardiovascular disease, and mortality [64].

### 4.2. Monocyte and Macrophage Senescence

Monocytes are particularly susceptible to the metabolic disturbances characteristic of MetS (Figure 5B). Hyperglycemia, oxidized LDL, saturated fatty acids, and adipose tissue-derived cytokines induce metabolic reprogramming and persistent inflammatory activation that promote senescence-like phenotypes [33,65].

At the molecular level, TLR4 activation by free fatty acids and damage-associated molecular patterns stimulates NF-κB signaling, leading to increased expression of IL-6, TNF-α, MCP-1, and other SASP components [45]. Simultaneously, mitochondrial ROS activate the NLRP3 inflammasome, resulting in caspase-1 activation and enhanced secretion of IL-1β and IL-18 [42,43].

Monocytes isolated from individuals with obesity and type 2 diabetes display increased expression of p16INK4a, p21CIP1, SA-β-gal activity, mitochondrial dysfunction, and DNA damage markers [21]. Moreover, chronic metabolic stress induces epigenetic reprogramming, leading to trained immunity, a persistent pro-inflammatory state characterized by sustained cytokine production even after removal of the initial stimulus [48].

Within adipose tissue, senescent macrophages contribute significantly to metaflammation through continuous SASP secretion. These cells impair insulin signaling through TNF-α-mediated inhibition of IRS-1 and promote adipocyte dysfunction, thereby reinforcing insulin resistance and metabolic deterioration (Figure 6) [33].

### 4.3. Neutrophil Senescence

Neutrophils are increasingly recognized as important participants in metabolic inflammation and immunosenescence. Chronic metabolic stress induces substantial changes in neutrophil function, phenotype, and lifespan (Figure 5C) [66,67].

Hyperglycemia and elevated free fatty acids increase neutrophil ROS production through activation of NADPH oxidase and mitochondrial oxidative pathways. Excessive ROS generation promotes DNA damage, activation of stress-response kinases, and functional exhaustion [68]. Furthermore, MetS is associated with enhanced NETosis, a process involving release of neutrophil extracellular traps (NETs), which contribute to endothelial dysfunction, thrombosis, and atherosclerotic plaque instability [67].

Recent evidence suggests that aging neutrophils exhibit altered CXCR4/CXCR2 signaling, impaired phagocytosis, dysregulated circadian trafficking, and increased inflammatory activity [69]. Persistent activation of NF-κB and NLRP3 pathways contributes to maintenance of these dysfunctional phenotypes (Figure 6). Although classical senescence markers are less studied in neutrophils than in proliferative cells, senescence-like changes clearly contribute to chronic inflammation and vascular injury in metabolic disease.

### 4.4. T-Cell Senescence

T lymphocytes represent one of the best-characterized examples of immunosenescence (Figure 5D). Chronic antigenic stimulation, oxidative stress, mitochondrial dysfunction, and inflammatory signaling promote progressive T-cell aging during both chronological aging and metabolic disease [70,71].

A key mechanism underlying T-cell senescence is repeated antigen-driven proliferation leading to telomere attrition and activation of DDR pathways. Telomere dysfunction activates ATM-dependent signaling and induces p53/p21-mediated growth arrest [72]. Senescent T cells also exhibit increased p38 MAPK activity, impaired DNA repair capacity, reduced autophagy, and persistent mTOR activation [73].

Phenotypically, senescent T cells lose expression of CD28 while gaining expression of CD57 and KLRG1. These cells display impaired proliferation but maintain high inflammatory activity, producing TNF-α, IFN-γ, and other SASP-like mediators [70]. Obesity and insulin resistance accelerate accumulation of CD8+CD28− and CD57+ T-cell populations in both peripheral blood and adipose tissue [72].

Metabolic dysfunction also alters T-cell immunometabolism. Reduced AMPK activity and chronic activation of the PI3K/Akt/mTOR pathway impair mitochondrial homeostasis and autophagic flux, promoting further senescence (Figure 6) [9,71]. These alterations contribute to defective immune surveillance, impaired vaccine responses, and persistent inflammation.

### 4.5. B-Cell Senescence

B-cell aging contributes substantially to the decline of adaptive immunity observed in aging and metabolic disease. Several studies have demonstrated that obesity accelerates B-cell senescence (Figure 5E) through mechanisms involving chronic inflammation, oxidative stress, and metabolic dysfunction [24].

Senescent B cells exhibit telomere shortening, mitochondrial dysfunction, impaired class-switch recombination, reduced antibody production, and increased secretion of inflammatory cytokines [25]. Activation of NF-κB signaling and suppression of autophagy appear to play central roles in the development of these phenotypes (Figure 6).

Aging and obesity are associated with expansion of age-associated B cells (ABCs), characterized by expression of T-bet, CD11c, and pro-inflammatory cytokines including TNF-α and IL-6 [25]. These cells contribute to systemic inflammation and may exacerbate insulin resistance through cytokine-mediated effects on adipose tissue and skeletal muscle.

Emerging evidence further suggests that SASP factors derived from senescent adipocytes and immune cells can induce secondary senescence in B-cell populations, highlighting the interconnected nature of senescence propagation within the immune system [21].

### 4.6. Natural Killer Cell Senescence

Natural killer (NK) cells are essential mediators of immune surveillance and represent one of the primary mechanisms responsible for senescent cells clearance. Consequently, dysfunction of NK cells has profound implications for senescence accumulation and chronic inflammation (Figure 5F) [74].

MetS is associated with reduced NK-cell cytotoxicity, impaired metabolic fitness, mitochondrial dysfunction, and diminished production of perforin and granzyme B [75]. Increased lipid accumulation within NK cells suppresses mTOR signaling and interferes with cellular metabolism, thereby impairing their capacity to eliminate senescent cells effectively.

Recent studies suggest that chronic inflammatory exposure induces features of exhaustion and senescence in NK cells, including altered mitochondrial dynamics, reduced proliferative capacity, and impaired cytokine production [76,77]. Consequently, clearance of senescent cells becomes less efficient, facilitating accumulation of SASP-producing cells throughout multiple tissues (Figure 6).

### 4.7. Platelet and Megakaryocyte Senescence

Although platelets lack nuclei and cannot undergo classical replicative senescence, they display age-associated functional alterations that contribute significantly to cardiometabolic disease (Figure 5G). Platelets derived from individuals with obesity and MEtS exhibit increased oxidative stress, mitochondrial dysfunction, hyperreactivity, and enhanced thrombotic potential [78].

Inflammatory cytokines and SASP components influence megakaryocyte maturation and platelet production within the bone marrow. Chronic activation of NF-κB signaling and oxidative stress pathways promotes generation of hyperreactive platelets capable of amplifying inflammatory responses through interactions with monocytes, neutrophils, and endothelial cells [78].

Activated platelets release numerous inflammatory mediators, extracellular vesicles, and mitochondrial components that contribute to vascular inflammation and thrombosis (Figure 6). These processes are particularly relevant in MEtS, where platelet hyperactivity is strongly associated with increased cardiovascular risk and adverse clinical outcomes [21].

## 5. Blood-Cell Senescence and Inflammaging

Inflammaging refers to the chronic, sterile, low-grade inflammatory state that develops during aging and contributes to the pathogenesis of age-related metabolic, cardiovascular, and immune disorders [79,80]. In the hematopoietic system, inflammaging is closely linked to the accumulation of senescent immune cells, which remain metabolically active despite impaired proliferative and functional capacity. Rather than being inert, senescent blood cells acquire a pro-inflammatory secretory profile that reshapes the systemic immune environment and promotes persistent inflammatory signaling.

A central mechanism through which senescent immune cells contribute to inflammaging is SASP. The SASP consists of a broad repertoire of soluble and extracellular factors, including pro-inflammatory cytokines, chemokines, growth factors, matrix-remodeling enzymes, extracellular vesicles, and damage-associated molecular patterns [81,82]. Among the most prominent SASP mediators are interleukin (IL)-1β, IL-6, IL-8/CXCL8, tumor necrosis factor-alpha (TNF-α), CCL2/MCP-1, granulocyte–macrophage colony-stimulating factor (GM-CSF), matrix metalloproteinases (MMPs), and reactive oxygen species (ROS) [40]. These factors can act locally to reinforce senescence in an autocrine manner while simultaneously inducing senescence and inflammatory activation in neighboring cells through paracrine signaling, a phenomenon known as the “bystander effect” [83].

In blood and immune cells, SASP production is driven by persistent activation of stress-response pathways. DNA damage responses, telomere dysfunction, mitochondrial ROS generation, cytosolic DNA sensing, NF-κB activation, p38 MAPK signaling, and inflammasome activation converge to induce inflammatory gene expression [35,45]. Mitochondrial dysfunction is particularly important because damaged mitochondria release mitochondrial DNA (mtDNA) and other mitochondrial danger-associated molecular patterns (mtDAMPs), which activate innate immune sensors and amplify cytokine production [35,84]. Consequently, senescent immune cells function not only as targets of cellular stress but also as active amplifiers of inflammatory responses.

Monocytes and macrophages are major contributors to blood-cell-derived inflammaging. Aging and metabolic stress promote the acquisition of a senescence-like phenotype characterized by increased secretion of IL-1β, IL-6, TNF-α, CCL2, and ROS, accompanied by impaired phagocytic function and defective resolution of inflammation [17,65]. In MetS, hyperglycemia, elevated free fatty acids, adipose tissue dysfunction, and oxidative stress further stimulate monocyte activation and macrophage senescence, thereby sustaining chronic low-grade inflammation in circulation and peripheral tissues [21].

Senescent T lymphocytes also contribute substantially to inflammaging. These cells exhibit reduced proliferative capacity, telomere shortening, loss of the co-stimulatory molecule CD28, increased expression of senescence-associated markers such as CD57 and KLRG1, and altered cytokine secretion profiles [70,85]. Despite impaired antigen responsiveness, senescent T cells often remain highly pro-inflammatory and produce elevated levels of interferon-gamma (IFN-γ), TNF-α, and IL-6 [81]. Their accumulation contributes to impaired immune surveillance, diminished vaccine responsiveness, and persistent inflammatory signaling [86].

B lymphocytes also undergo age-related functional remodeling that contributes to inflammaging. Senescent B cells display reduced antibody production, impaired class-switch recombination, diminished responsiveness to antigenic stimulation, and increased secretion of pro-inflammatory cytokines, collectively contributing to dysregulated humoral immunity [25,87]. Similarly, aged neutrophils exhibit impaired chemotaxis, phagocytosis, and microbial killing but increased ROS generation and enhanced formation of neutrophil extracellular traps (NETs), processes that can exacerbate tissue damage and vascular inflammation [88].

Importantly, blood-cell senescence and inflammaging reinforce one another through self-perpetuating feed-forward loops. Chronic inflammation accelerates oxidative stress, DNA damage, mitochondrial dysfunction, and telomere attrition in hematopoietic and immune cells, thereby promoting additional senescence [71]. In turn, senescent immune cells release SASP factors that sustain systemic inflammation and recruit additional immune cells into inflammatory circuits [89]. This bidirectional relationship is particularly relevant in MetS, where nutrient excess, insulin resistance, adipose tissue inflammation, and vascular dysfunction establish a persistent inflammatory milieu that accelerates immune-cell aging [21].

The inflammatory mediators produced by senescent blood cells have profound systemic consequences. IL-6 and TNF-α interfere with insulin signaling, promote hepatic acute-phase responses, and contribute to endothelial activation and vascular dysfunction [84]. IL-1β enhances inflammasome-driven inflammation and may impair pancreatic β-cell function, thereby exacerbating glucose dysregulation [85,90]. Chemokines such as CCL2 facilitate monocyte recruitment into adipose tissue and atherosclerotic lesions, while MMPs contribute to extracellular matrix remodeling and plaque instability [91]. Through these mechanisms, senescent immune cells provide a mechanistic link between cellular aging, chronic systemic inflammation, insulin resistance, atherosclerosis, and cardiovascular disease.

Overall, blood-cell senescence represents both a consequence and a driver of inflammaging. Through SASP production, mitochondrial dysfunction, impaired immune regulation, and altered metabolic programming, senescent immune cells perpetuate chronic inflammation and contribute to the progression of metabolic and cardiovascular disease. Consequently, therapeutic strategies targeting senescent immune cells or modulating their inflammatory secretome may represent promising approaches to attenuate inflammaging and its adverse metabolic consequences.

## 6. BCs Senescence as a Biomarker

The growing recognition of cellular senescence as a fundamental mechanism linking aging, chronic inflammation, and metabolic dysfunction has stimulated interest in the development of reliable senescence biomarkers. Among the various tissues affected by aging and metabolic disease, circulating blood cells represent an attractive source of biomarkers because they can be obtained through minimally invasive procedures and reflect both systemic and immune-specific alterations. Consequently, BCs senescence markers are increasingly being investigated as indicators of biological aging, cardiometabolic risk, and disease progression in individuals with MetS and related disorders [29,92].

### 6.1. Leukocyte Telomere Length

Leukocyte telomere length (LTL) is one of the most extensively studied biomarkers of biological aging and cellular senescence. Telomeres shorten progressively during cellular replication and are particularly vulnerable to oxidative stress and chronic inflammation, both of which are highly prevalent in MetS [35]. Numerous epidemiological studies have demonstrated that shortened LTL is associated with obesity, insulin resistance, T2DM, hypertension, and cardiovascular disease [38,93]. Meta-analyses have further shown that individuals with MetS exhibit significantly shorter leukocyte telomeres compared with metabolically healthy controls, supporting the concept that metabolic stress accelerates biological aging [39]. Although LTL lacks specificity for individual senescent cell populations, its strong association with age-related disease has established it as one of the most widely used systemic biomarkers of senescence.

### 6.2. Senescent T-Cell Phenotypes: CD28−CD57+ and KLRG1+ T Cells

Immunosenescence is characterized by the accumulation of highly differentiated T-cell populations that exhibit reduced proliferative capacity and altered effector functions. Among these, CD28−CD57+ T cells are considered one of the most robust biomarkers of T-cell senescence [70]. Loss of the co-stimulatory molecule CD28 and acquisition of CD57 are associated with telomere shortening, impaired proliferation, mitochondrial dysfunction, and increased production of pro-inflammatory cytokines such as TNF-α and IFN-γ [85,94]. Increased frequencies of CD28−CD57+ and KLRG1+ T cells have been reported in obesity, T2DM, and cardiovascular disease, where they correlate with insulin resistance, systemic inflammation, and adverse clinical outcomes [22,27]. Because these cells can be readily quantified by flow cytometry, they represent promising biomarkers of immune aging in metabolic disorders.

### 6.3. p16INK4a and p21CIP1 Expression

The cyclin-dependent kinase inhibitors p16INK4a and p21CIP1 are among the most widely accepted molecular markers of cellular senescence. Both proteins contribute to irreversible cell-cycle arrest and are activated in response to DNA damage, oxidative stress, telomere dysfunction, and oncogenic signaling [32]. Expression of p16INK4a in peripheral blood T cells has been shown to increase exponentially with chronological age and has been proposed as a molecular indicator of biological aging [62]. Elevated p16INK4a and p21CIP1 expression has also been observed in circulating immune cells from individuals with obesity and T2DM, suggesting that metabolic stress accelerates senescence-associated molecular programs [33]. Importantly, p16INK4a expression in blood cells has been associated with frailty, physical dysfunction, and age-related disease burden, supporting its potential clinical utility [95].

### 6.4. Circulating Inflammatory Cytokines and SASP Factors

Senescent immune cells contribute to systemic inflammation through the production of SASP factors, making circulating inflammatory mediators attractive indirect biomarkers of senescence burden. Elevated serum concentrations of IL-6, TNF-α, IL-1β, MCP-1/CCL2, and C-reactive protein are consistently associated with aging, obesity, MetS, and T2DM [20,21]. Among these markers, IL-6 has emerged as one of the strongest predictors of age-related morbidity and mortality [96]. However, because inflammatory cytokines can originate from multiple cell types and pathological processes, they are generally considered complementary rather than definitive biomarkers of cellular senescence. Nevertheless, integrating cytokine measurements with cell-specific senescence markers may improve the assessment of senescence burden in clinical settings.

### 6.5. Peripheral Blood Mononuclear Cells (PBMCs) Transcriptomic Signatures

Advances in transcriptomic technologies have enabled the identification of senescence-associated gene expression signatures in PBMCs. Senescent PBMCs exhibit altered expression of genes involved in DNA damage responses, inflammatory signaling, mitochondrial function, cell-cycle regulation, and metabolic pathways [97]. Transcriptomic analyses have identified increased expression of *CDKN2A* (encoding p16INK4a), *CDKN1A* (encoding p21CIP1), inflammatory cytokines, NF-κB-related genes, and SASP-associated pathways in aged and metabolically compromised individuals [29]. More recently, single-cell RNA sequencing studies have revealed distinct senescent immune-cell subsets with unique transcriptional profiles that may not be detectable using conventional approaches [98]. These findings suggest that PBMC transcriptomic signatures could provide a comprehensive and sensitive assessment of blood-cell senescence, although standardization and validation across populations remain necessary before routine clinical implementation.

### 6.6. Clinical Relevance and Potential Implementation of Blood-Cell Senescence Biomarkers

From a clinical perspective, blood-cell senescence biomarkers could complement conventional metabolic and cardiovascular risk indicators by providing information on biological aging, immune dysfunction, and cumulative cellular stress. In patients with MetS, these biomarkers may potentially support the identification of individuals whose biological aging burden is greater than expected from chronological age, thereby refining risk stratification beyond established parameters such as body mass index, blood pressure, lipid profile, and glycemic status. Longitudinal assessment of senescence-associated markers could also help monitor disease progression and evaluate biological responses to weight loss, exercise, dietary modification, antidiabetic treatment, or future senescence-targeted interventions.

However, the clinical utility of these biomarkers remains investigational. Leukocyte telomere length, p16INK4a and p21CIP1 expression, senescence-associated T-cell phenotypes, circulating SASP mediators, and PBMC transcriptomic signatures each capture different and partly overlapping aspects of cellular aging. None is sufficiently specific or standardized to serve as an independent diagnostic or prognostic test for MetS. Their interpretation may be influenced by chronological age, sex, smoking, infections, medication use, obesity severity, comorbidities, and differences in cell composition and analytical methodology. Therefore, composite panels combining cell-specific phenotypes, molecular markers, functional measurements, and conventional clinical variables are likely to offer greater clinical value than any single biomarker.

Before implementation in routine practice, these panels require analytical standardization, definition of reference ranges and clinically meaningful cut-off values, and validation in large prospective cohorts representing diverse patient populations. Studies should also determine whether senescence biomarkers provide predictive information beyond established cardiometabolic risk scores and whether biomarker-guided interventions improve patient outcomes. At present, their most realistic applications are patient stratification in clinical research, monitoring of biological responses to interventions, and identification of individuals suitable for trials of senolytic or senomorphic therapies.

## 7. Gaps and Future Directions

Despite substantial progress in understanding the relationship between cellular senescence, immune dysfunction, and MetS, several important knowledge gaps remain as many mechanistic and translational questions remain unresolved. Addressing these limitations will be essential for establishing the clinical relevance of BCs senescence and for developing effective senescence-targeted interventions in metabolic disease.

Recent advances in single-cell technologies offer unprecedented opportunities to improve our understanding of BCs senescence. Traditional bulk analyses often obscure the substantial heterogeneity that exists within immune-cell populations. Single-cell RNA sequencing, single-cell epigenomic profiling, high-dimensional flow cytometry, and spatial transcriptomics can identify distinct senescent subpopulations and characterize their functional states at high resolution [98,99]. These approaches may reveal previously unrecognized senescence trajectories, cell-type-specific SASP signatures, and interactions between senescent and non-senescent immune cells. Moreover, integrating single-cell analyses with metabolomic and proteomic profiling could provide a more comprehensive understanding of how metabolic stress reshapes immune-cell aging.

A further priority is the establishment of causal relationships between immune-cell senescence and metabolic outcomes. Although in vitro studies and animal models suggest that senescent immune cells contribute to insulin resistance, adipose tissue inflammation, endothelial dysfunction, and atherosclerosis, definitive evidence in humans remains limited [31,33]. Most clinical data are observational or cross-sectional and therefore cannot exclude reverse causation or confounding by chronological age, obesity severity, medication use, comorbidities, and persistent infections. Moreover, differences between experimental systems and human disease—including species-specific immune biology, artificial senescence induction, supraphysiological exposures, and the heterogeneity of human MetS—limit direct extrapolation. Future investigations should combine longitudinal human cohorts with advanced genetic, pharmacological, and cell-specific approaches to determine whether senescence precedes metabolic deterioration and whether targeting senescent immune cells directly improves metabolic health. The emergence of senolytic and senomorphic therapies offers an opportunity to test causality by evaluating whether selective elimination or functional modulation of senescent BCs can reverse metabolic dysfunction and reduce chronic inflammation.

Another important area for future research involves the interaction between clonal hematopoiesis, immunosenescence, and metabolic disease. Emerging evidence suggests that CHIP and cellular senescence may share common mechanisms involving DNA damage, chronic inflammation, and hematopoietic stem-cell dysfunction [53]. However, the extent to which CHIP promotes immune-cell senescence or vice versa remains poorly understood. Longitudinal studies integrating genomic, immunological, and metabolic data may help clarify these complex relationships.

Future studies should also address population diversity and biological variability. Many existing investigations have focused on older adults from specific geographic or ethnic backgrounds, limiting the generalizability of findings. Sex-specific differences, genetic susceptibility, lifestyle factors, dietary patterns, and environmental exposures may all influence immune-cell senescence and metabolic disease progression [100]. Larger and more diverse cohorts will therefore be necessary to identify population-specific determinants of BCs senescence and to support the development of personalized therapeutic strategies.

One of the major limitations of the current literature is the predominance of cross-sectional studies. Most human investigations have evaluated senescence markers at a single time point, making it difficult to determine whether BCs senescence is a cause, consequence, or merely a correlate of metabolic dysfunction [21,32]. Longitudinal cohort studies are therefore needed to characterize the temporal dynamics of immune-cell senescence during the development and progression of MetS. Such studies could clarify whether senescence-related alterations precede the onset of insulin resistance, obesity, and cardiovascular complications or arise secondary to established metabolic abnormalities. Furthermore, prospective studies could evaluate whether senescence biomarkers predict disease progression, therapeutic response, or cardiovascular outcomes independently of traditional risk factors.

Another significant challenge is the lack of standardized and universally accepted biomarkers of BCs senescence. Cellular senescence is a heterogeneous and multifaceted process, and no single marker reliably identifies senescent cells across all immune-cell populations. Commonly used markers—including p16INK4a, p21CIP1, senescence-associated β-galactosidase (SA-β-gal), telomere length, γH2AX, and SASP factors—often capture only specific aspects of the senescent phenotype and may vary according to cell type, disease state, and experimental conditions [29]. Future studies should focus on establishing standardized panels of BCs senescence markers that combine molecular, functional, and phenotypic characteristics. The development of reproducible and clinically applicable assays will be critical for comparing results across studies and for translating senescence research into clinical practice.

Finally, the translational potential of BCs senescence research remains largely unexplored. Because circulating immune cells are readily accessible through minimally invasive blood sampling, they represent attractive candidates for biomarker development and therapeutic monitoring. Future clinical studies should evaluate whether BCs senescence signatures can serve as early indicators of metabolic dysfunction, predictors of cardiovascular risk, or biomarkers of response to lifestyle interventions, antidiabetic therapies, and emerging senescence-targeted treatments.

In summary, while substantial evidence links BCs senescence to chronic inflammation and metabolic dysfunction, important challenges remain regarding biomarker standardization, mechanistic understanding, and clinical translation. Future longitudinal studies, single-cell approaches, and interventional investigations targeting senescent immune cells will be essential for establishing causality and determining the therapeutic potential of modulating immune-cell senescence in MetS.

## 8. Conclusions

MEtS is characterized by a complex interplay of metabolic, inflammatory, and immunological disturbances that collectively accelerate biological aging. Growing evidence indicates that BCs senescence is a central component of this process, linking metabolic stress to chronic inflammation, immune dysfunction, and cardiovascular complications. Oxidative stress, mitochondrial dysfunction, telomere attrition, DNA damage, epigenetic alterations, and clonal hematopoiesis contribute to the development of senescent phenotypes in hematopoietic stem cells and mature immune-cell populations. These senescent cells actively participate in disease progression through the production of pro-inflammatory mediators and the establishment of a senescence-associated secretory phenotype, thereby reinforcing inflammaging and metabolic dysfunction.

Importantly, BCs senescence is not merely a marker of biological aging but appears to be an active driver of metabolic and cardiovascular pathology. Advances in high-throughput and single-cell technologies have greatly expanded our understanding of the cellular and molecular mechanisms underlying immune-cell senescence, while emerging senescence-targeted therapies offer promising opportunities for intervention. Nevertheless, significant challenges remain regarding the identification of reliable biomarkers, the establishment of causal relationships, and the translation of experimental findings into clinical practice.

A deeper understanding of the mechanisms governing BCs senescence may provide novel insights into the pathogenesis of MetS and facilitate the development of innovative diagnostic and therapeutic strategies. Ultimately, targeting senescent immune cells and their inflammatory secretome may represent a promising approach to reduce chronic inflammation, improve metabolic health, and mitigate the burden of age-related cardiometabolic disease.

## Data Availability

No new data were created or analyzed in this study. Data sharing is not applicable to this article.

## Abbreviations

The following abbreviations are used in this manuscript:

AGEs	Advanced glycation end products
Akt	Protein kinase B
AMPK	AMP-activated protein kinase
ASC	Apoptosis-associated speck-like protein containing a CARD
ATM	Ataxia-telangiectasia mutated
ATP	Adenosine triphosphate
ATR	ATM- and Rad3-related
CARD	Caspase activation and recruitment domain
CCL2	C–C motif chemokine ligand 2
CD11c	Cluster of differentiation 11c; integrin alpha X
CD28	Cluster of differentiation 28
CD40L	CD40 ligand
CD57	Cluster of differentiation 57
CDK	Cyclin-dependent kinase
CDK4	Cyclin-dependent kinase 4
CDK6	Cyclin-dependent kinase 6
CHK1	Checkpoint kinase 1
CHK2	Checkpoint kinase 2
CVD	Cardiovascular disease
CXCL8	C–X–C motif chemokine ligand 8
CXCR2	C–X–C chemokine receptor 2
CXCR4	C–X–C chemokine receptor 4
DDR	DNA damage response
DNA	Deoxyribonucleic acid
E2F	E2F transcription factor
ERK	Extracellular signal-regulated kinase
FAO	Fatty acid oxidation
FFA	Free fatty acids
G1	Gap 1 phase of the cell cycle
GM-CSF	Granulocyte–macrophage colony-stimulating factor
HSCs	Hematopoietic stem cells
IFN-γ	Interferon gamma
IKK	Inhibitor of nuclear factor kappa B kinase
IκBα	Inhibitor of nuclear factor kappa B alpha
IL	Interleukin
IL-1β	Interleukin-1 beta
IL-1R	Interleukin-1 receptor
IL-6	Interleukin-6
IL-8	Interleukin-8
IL-18	Interleukin-18
INK4a	Inhibitor of cyclin-dependent kinase 4a
IRS-1	Insulin receptor substrate 1
JAK	Janus kinase
JNK	c-Jun N-terminal kinase
KLRG1	Killer cell lectin-like receptor subfamily G member 1
LDL	Low-density lipoprotein
LRR	Leucine-rich repeat
MAPK	Mitogen-activated protein kinase
MASLD	Metabolic dysfunction-associated steatotic liver disease
MCP-1	Monocyte chemoattractant protein 1; also designated CCL2
MetS	Metabolic syndrome
MiDAS	Mitochondrial dysfunction-associated senescence
MMPs	Matrix metalloproteinases
mtDAMPs	Mitochondrial damage-associated molecular patterns
mtDNA	Mitochondrial DNA
mTOR	Mechanistic target of rapamycin
NACHT	NAIP, CIITA, HET-E and TP1 domain
NADPH	Reduced nicotinamide adenine dinucleotide phosphate
NAFLD	Non-alcoholic fatty liver disease
NETs	Neutrophil extracellular traps
NETosis	Neutrophil extracellular trap formation
NF-κB	Nuclear factor kappa B
NK	Natural killer
NLRP3	NOD-, LRR- and pyrin domain-containing protein 3
NOX2	NADPH oxidase 2
ox-LDL	Oxidized low-density lipoprotein
P	Phosphorylation
p10	10 kDa catalytically active cleavage subunit of caspase-1
p16INK4a	Cyclin-dependent kinase inhibitor 2A; p16 inhibitor of cyclin-dependent kinase 4a
p20	20 kDa catalytically active cleavage subunit of caspase-1
p21CIP1	Cyclin-dependent kinase inhibitor 1A
p38 MAPK	p38 mitogen-activated protein kinase
p50	Nuclear factor kappa B subunit 1
p53	Tumor protein p53
p65	RelA proto-oncogene/NF-κB subunit
PAI-1	Plasminogen activator inhibitor 1
PI3K	Phosphoinositide 3-kinase
PYD	Pyrin domain
Rb	Retinoblastoma protein
ROS	Reactive oxygen species
SA-β-gal	Senescence-associated beta-galactosidase
SASP	Senescence-associated secretory phenotype
STAT	Signal transducer and activator of transcription
T-bet	T-box transcription factor TBX21
TGF-β	Transforming growth factor beta
TLR	Toll-like receptor
TLR4	Toll-like receptor 4
TLR9	Toll-like receptor 9
TNF-α	Tumor necrosis factor alpha
T2DM	Type 2 diabetes mellitus
Ub	Ubiquitination
γH2AX	Phosphorylated histone H2AX
